# Expression of Chitotriosidase in Macrophages Modulates Atherosclerotic Plaque Formation in Hyperlipidemic Mice

**DOI:** 10.3389/fphys.2020.00714

**Published:** 2020-06-23

**Authors:** Jonathan Yap, Sara McCurdy, Martin Alcala, Jason Irei, Jan Garo, Whitney Regan, Bog-Hieu Lee, Shiro Kitamoto, William A. Boisvert

**Affiliations:** ^1^Center for Cardiovascular Research, John A. Burns School of Medicine, University of Hawaii, Honolulu, HI, United States; ^2^Department of Chemistry and Biochemistry, Facultad de Farmacia, Universidad CEU San Pablo, Madrid, Spain; ^3^Department of Food and Nutrition, School of Food Science and Technology, Chung-Ang University, Seoul, South Korea; ^4^Departments of Cardiovascular Medicine and Advanced Therapeutics for Cardiovascular Diseases, Graduate School of Medical Sciences, Kyushu University, Fukuoka, Japan

**Keywords:** atherosclerosis, macrophage, chitotriosidase, hyaluronic acid, collagen

## Abstract

**Objective:**

To determine whether overexpression of the chitin degrading enzyme, chitotriosidase (CHIT1), modulates macrophage function and ameliorates atherosclerosis.

**Approach and Results:**

Using a mouse model that conditionally overexpresses CHIT1 in macrophages (CHIT1-Tg) crossbred with the *Ldlr*^–/–^ mouse provided us with a means to investigate the effects of CHIT1 overexpression in the context of atherosclerosis. *In vitro*, CHIT1 overexpression by murine macrophages enhanced protein expression of IL-4, IL-8, and G-CSF by BMDM upon stimulation with a combination of lipopolysaccharide (LPS) and interferon-γ (IFN-γ). Phosphorylation of ERK1/2 and Akt was also down regulated when exposed to the same inflammatory stimuli. Hyperlipidemic, *Ldlr*^–/–^-CHIT1-Tg (CHIT1-OE) mice were fed a high-fat diet for 12 weeks in order to study CHIT1 overexpression in atherosclerosis. Although plaque size and lesion area were not affected by CHIT1 overexpression *in vivo*, the content of hyaluronic acid (HA) and collagen within atherosclerotic plaques of CHIT1-OE mice was significantly greater. Localization of both ECM components was markedly different between groups.

**Conclusions:**

These data demonstrate that CHIT1 alters cytokine expression and signaling pathways of classically activated macrophages. *In vivo*, CHIT1 modifies ECM distribution and content in atherosclerotic plaques, both of which are important therapeutic targets.

## Introduction

Chitin is among the most abundant biopolymers in nature, second only to cellulose. It is a linear polysaccharide made up of repeating *N*-acetyl-D-glucosamine (GlcNAC) monomers. Chitin functions as the primary structural component in the exoskeleton of arthropods, and is produced by mollusks, crustaceans, fungi, and nematodes as well (Tharanathan and Kittur, 2003). Endogenous chitin is not present in vertebrates and is, in fact, recognized by macrophage TLRs as a pathogen associated molecular pattern (PAMP). Like most mammalian chitinases, Chitotriosidase (CHIT1) is a member of the glycosyl-hydrolase enzymatic family 18 (GH18). Cleavage of substrate is achieved through hydrolysis of β(1→4) glycosidic bonds. There are two major isoforms of CHIT1 that arise from post-translational modification. In macrophages, CHIT1 is initially synthesized as a 50 kDa protein. This isoform is either secreted in response to an inflammatory stimulus or packaged in lysosomes and lysosome-related organelles (LRO) where the acidic environment within is believed to promote cleavage into a still fully enzymatically active 39 kDa isoform (Renkema et al., 1995, 1997).

Several different reports have described elevated serum CHIT1 activity in atherosclerotic patients and animals (Artieda et al., 2003; Fach et al., 2004; Karadag et al., 2008). Previously published data from our group showed that CHIT1 inhibition using a chitinase inhibitor, allosamidin, promotes atherosclerosis in ApoE^–/–^ mice (Kitamoto et al., 2013). Pretreatment of bone marrow-derived macrophages (BMDM) *in vitro* with allosamidin and subsequent stimulation with IFN-γ significantly upregulated pro-inflammatory mediators associated with M1 macrophage polarization such as MCP-1, TNF-α, iNOS, IL-6, IL-12, and IL-1β when compared to IFN-γ treatment alone. *In vivo*, ApoE^–/–^ mice fed a Western diet for 6 weeks and administered allosamidin via a surgically implanted pump, exhibited significantly greater lesion area in the aortic arch compared to vehicle control. Moreover, we found that lesion area was also significantly larger in the aortic sinus, and that macrophage deposition was also significantly greater.

Because we observed an exacerbation of atherosclerosis by inhibiting CHIT1, we sought to examine if overexpression of CHIT1 in macrophages may exhibit possible athero-protective properties. Our findings demonstrate that CHIT1 overexpression *in vitro* augmented the transcription and expression of cytokines and chemokines associated with atherosclerosis. We also observed differences in plaque morphology between atherosclerotic mice overexpressing CHIT1 compared to littermate controls. Given these findings and given that chitin does not exist in atherosclerotic plaques, further exploration of the non-traditional role of CHIT1 in plaque macrophages may be warranted and may indeed provide novel insight into macrophage behavior and atherosclerotic plaque morphology.

## Materials and Methods

### Mouse Strains

**FIGURE 1:**
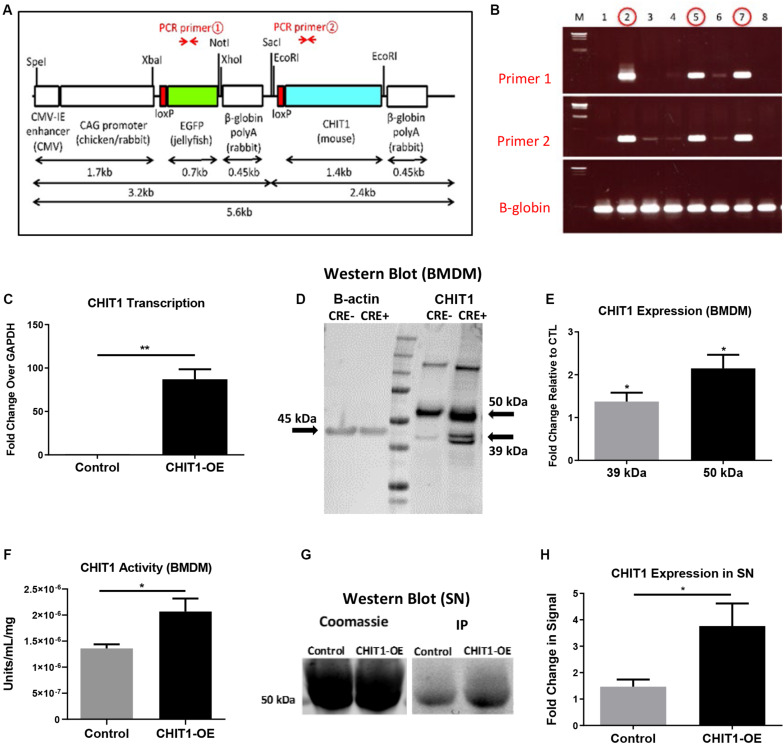
**(A)** The transgene was designed with an EGFP stop sequence (green) that is removed in the presence of Cre recombinase. When Cre recombinase is expressed in lysozyme producing cells, the CHIT1 gene (blue) is constitutively expressed by macrophages. **(B)** Results from genotyping F0 mice indicate the presence of both primers and confirm transgene insertion. B -globin gene was used as internal control. **(C)** Transcription of CHIT1 mRNA was quantified using qPCR. cDNA was isolated from control and CHIT1-OE BMDM (*P* = 0.0059). **(D)** Western blot imaging of CHIT1 protein expression. Both the 50 kDa and 39–42 kDa isoforms were significantly increased in BMDM from CHIT1-OE mice compared to controls (*P* = 0.0245 and 0.0168 respectively). **(E)** Membranes after Western blot were imaged using Licor Odyssey CLx and analyzed with image studio software measurements reflect the fold change in florescence intensity. **(F)** CHIT1 activity was analyzed in BMDM from control (Cre-) and CHIT1-OE (Cre+). Liberated 4-MU emission was measured with a plate reader to determine enzymatic activity (*P* = 0.0305. **(G)** immunoprecipitation was performed on supernatants of BMDM harvested from CHIT1-OE mice and control animals. Coomassie staining of the membrane was used as a total protein control (*P* = 0.0330). **(H)** Analysis of Western blot images with image studio software showed a significant increase in CHIT1 protein expression. *N* = 3; *P<0.05; **P<0.01.

Wild-type C57BL/6 mice and LDLR null mice generated on a C57BL/6 background animal were purchased from Jackson Laboratories (Bar Harbor, Maine). CHIT1-OE mice were developed in cooperation with Kyushu University and Riken research Institute (Saitama, Japan). Briefly, the transgene depicted in Figure 1A was microinjected into fertilized mouse embryos.

To confirm successful transgene insertion, PCR and gel electrophoresis was used to detect the presence of primer sequences specific to the CHIT1 and EGFP sections of the transgene (Figure 1B). Ldlr^–/–^ mice were crossbred with the CHIT1-Tg mice to produce Ldlr^–/–^-CHIT1-Tg animals, which overexpress CHIT1 and are prone to atherosclerosis. Given that the EGFP stop sequence is flanked by loxp sites (floxed), we employed a Cre-lox method of overexpression. For our experiments, floxed Ldlr^–/–^-CHIT1-Tg mice were used as littermate controls. Experimental animals were produced by breeding Ldlr^–/–^-CHIT1-Tg mice with LysM-CRE mice, in which Cre-recombinase is under transcriptional control of the lysozyme promoter. Because lysozyme is constitutively produced by macrophages, the resultant animals overexpress CHIT1 as the floxed EGFP/β-globin, poly-A stop sequence is excised. Thus, the genetic characterization of our experimental mouse models can be described as follows: ldlr^–/–^- CHIT1-Tg-LysM^Cre^ (CHIT1-OE) Mice between 8 and 12 weeks of age were used for isolation of bone marrow derived macrophages. Male CHIT1-OE mice between 8 and 10 weeks were utilized for the in vivo atherosclerosis study. At 8–10 weeks of age, animal subjects were placed on HFD containing 15.8% (wt/wt) fat and 1.25% cholesterol (wt/wt) (diet 94059; Harlan Teklad Laboratories, Indianapolis, IN, United States) for 12 weeks to induce atherosclerosis. All animal studies and protocols were approved by the University of Hawaii Institutional Animal Care and Use Committee.

### Bone Marrow-Derived Macrophage Cell Isolation and *in vitro* Cell Culture

Experimental and control mice were sacrificed by CO_2_ asphyxiation at 8–10 weeks of age. 70% EtOH was sprayed on the animals to disinfect the skin. Each femur and tibia was surgically removed under a biosafety hood. Using a 30-gauge needle, the marrow within the exposed bones were flushed out with ice cold PBS into a 50-mL falcon tube while on ice. The isolated bone marrow was broken up by vigorous pipetting with a 10-mL pipette. It was then passed through a 40-μm cell strainer into a 50-mL falcon tube, and centrifuged at 1,300 RPM (≈300 × *g*) for 5 min to form a pellet. PBS was aspirated and the remaining pellet was resuspended in BMDM differentiation medium (DMEM/F12, [Gibco], 10% FBS, 20% L929 conditioned medium, and 1% pen/strep [Gibco]). Cell viability was determined by staining a 10 μL sample of cells in suspension with trypan blue. Cells were cultured at a density of 1 × 10^6^ cells per mL and plated in 15-cm tissue-culture treated plates (Corning) with 25 mL of medium. 5–10 mL of media was added to the plate every other day for 7 days. Differentiated macrophages adherent to the plate were detached using non-enzymatic Cell Stripper solution (Gibco). The cells were then counted and re-plated as specified in each experiment.

### Preparation of Lysates From Cells and Tissues

RIPA buffer (10 mM Tris-Cl [pH 7.6], 1 mM EDTA, 1% Triton X-100, 0.1% sodium deoxycholate, 0.1% SDS, 140 mM NaCl) and 1X Halt protease and phosphatase inhibitor cocktails (Thermo Fisher) was used in preparation of lysates. Adherent cells in 6-well plates were washed with PBS which was then aspirated from each well before adding 100 μL of ice cold lysis buffer with protease/phosphatase inhibitor cocktails (diluted 1:1,000) (Roche). Plates were kept on ice for 15 min before scraping the wells with a cell scraper and transferring the lysates to 1.5 ml Eppendorf tubes on ice. Samples were then sonicated by pulsing 10 times with a probe sonicator (Thermo Fisher) at power level 2.5, and centrifuged at 400 × *g* for 5 min at 4°C. Lysates were transferred to a new Eppendorf tube and the pelleted cell debris was discarded. Pierce BCA protein assay kit (Thermo Fisher) was used to determine protein concentrations of lysates in a 1:10 dilution of samples.

### Polyacrylamide Gel Electrophoresis and Western Blot Transfer

Precast NuPAGE^®^ Novex^®^ 4–12% Bis-Tris Mini or Midi protein gels (Invitrogen) with 12 or 20-lanes respectively, were used in conjunction with the appropriate mini or midi electrophoresis gel box. 4× SDS loading buffer (0.1 M Tris-HCl pH 6.8, 0.7% SDS, 33% glycerol, 0.01% bromophenol blue, 8% β-mercaptoethanol) was added to 10–20 μg of protein and this mixture was heated and maintained at 95°C for 5 min. Samples were loaded into each lane and 4 μL of Li-cor 1-color protein marker was added as a size reference latter. Gels were run in 1X NuPAGE^®^ MES SDS Running Buffer (Invitrogen) at a constant voltage of 150 V until the loading dye has completely migrated to the bottom of the gel. The gel is removed from its casing and placed on a low florescence PVDF membrane that has been activated in 100% methanol and immersed in 1× Efficient^TM^ Western Transfer Buffer (G-Biosciences^®^ #786-019) with 20% methanol for 5 min. The gel and membrane together were placed between sheets of filter paper soaked in transfer buffer, and compressed tightly within the transfer cassette. The transfer cassette was placed in the transfer chamber along with an ice pack and stir Rod. The chamber was then put on ice. Protein was transferred to the membrane after running for 1 h at 100 V.

### Membrane Incubation With Antibodies and Scanning

The membranes were blocked in 1:1 Li-cor blocking buffer and PBS for 1 h with gentle agitation on an orbital shaker. The blocked membranes were incubated with primary antibodies (Table 1) diluted in Li-cor blocking buffer at room temperature for 1 h or at 4°C overnight. Membranes were subsequently washed three times with 0.1% Tween20 in PBS. Secondary antibodies diluted in Li-cor blocking buffer were incubated with membranes at room temperature for 1 h in the dark. After washing three times for 5 min each with 0.1% PBST, membranes were scanned with a Li-cor Odyssey infrared scanner and images were analyzed with Li-cor Image Studio (Li-cor Biosciences).

**TABLE 1:** List of antibodies used in Western Blot and IHC.

Name	Product number	Company	Dilution
CHIT1	sc-271460	Santa Cruz Biotechnology	1:500
MOMA	sc-59332	Santa Cruz Biotechnology	1:500
β-actin	ABT264	Sigma	1:2,000
ERK1/2	9102	Cell signaling	1:1,000
pERK1/2 (Thr202/Tyr204)	9101	Cell signaling	1:1,000
pAKT (Ser473)	4060	Cell signaling	1:1,000
PIκB-α (Ser32)	2859	Cell signaling	1:1,000
IRDye 800CW	925-32213	Licor	1:10,000
IRDye 800CW	926-32214	Licor	1:10,000
IRDye 680CW	926-68072	Licor	1:10,000

### Reverse Transcriptase Quantitative PCR (RT-qPCR)

Total RNA was extracted by adding 500 μL–1 mL TRIzol reagent (Life Technologies) to each sample in a 1.5 mL Eppendorf tube. The sample was then vortexed until completely dissolved and chloroform was added to TRIzol in a 20% v/v ratio, and inverted vigorously to mix. The samples were centrifuged at 12,400 RPM for 15 min in a pre-cooled centrifuge at 4°C. Once phase separation occurs, the aqueous fraction was carefully pipetted into a separate tube and combined with an equal volume of nuclease-free, 70% ethanol. Samples were mixed thoroughly and put through RNA purification columns provided in the Qiagen RNeasy RNA extraction kit. DNA digest was performed, on-column, for 15 min at room temperature. After the appropriate wash steps, columns were allowed to completely dry. Elution of RNA from the columns was performed using 30–50 μL nuclease-free water. RNA concentration and quality was determined with a NanoDrop 2000. RNA was used immediately for RT-qPCR or stored at −80°C. 1 μg of total RNA per sample would be used to transcribe cDNA using the qScript cDNA synthesis kit (Quanta Biosciences). 80 μL of nuclease-free water was added to each cDNA sample, bringing the total volume to 100 μL. In performing the RT-qPCR, 4 μL of cDNA was added to each well along with a master mix of 12.5 μL SYBR Green 2X master mix containing ROX (Roche), 0.5 μL of both forward and reverse primers (10 μM), and 7 μL of nuclease-free water. 10 μL of the reaction mixture was pipetted in triplicate to a 384-well qPCR plate and analyzed with an Applied Biosystems 7900HT Fast Real-Time PCR System.

### Cytokine Array

The Proteome Profiler Array: Mouse Chemokine Array Kit (R&D) was used to determine relative levels of 40 different mouse Cytokines and Chemokines. Cell lysates were prepared by rinsing BMDM cultures with PBS and then adding 100–150 μL of lysis buffer to each well. The cells and lysis buffer are incubated for 30 min before harvesting with a cell scraper. Protein concentration was determined using BCA assay. 2 mL of array buffer 6 (blocking buffer) is added to each well and the pre-blotted, nitrocellulose membranes that were provided in the kit are added to the wells. The membranes were incubated for 1 h at room temperature. 15 μL of reconstituted Mouse Cytokine Array Panel A Detection Antibody Cocktail was added to each sample containing 100 μg total protein. The wells were emptied of Array Buffer 6 and the samples/antibody mixtures were added to the wells and allowed to incubate overnight at 2–8°C on a rocking platform shaker. Each membrane was washed and then 2 mL of IRDye 800CW Streptavidin (LI-COR, Catalog #926-32230) diluted 1:2,000 using the Array Buffer 6 was added to each well of the dish. After incubating for 30 min at room temperature, each membrane was removed and images were collected with an Odyssey CLx imaging system.

### Blood Collection and Sacrifice of Study Animals

Blood from each mouse was collected at the start and end point of the study using a submandibular bleeding technique. A portion of the whole blood collected was centrifuged at 1,500 × *g* for 15 min to achieve plasma separation. Plasma samples were used to measure lipid levels. At the study’s conclusion, animals were euthanized by CO_2_ asphyxiation and total blood was collected through cardiac puncture of the left ventricle. Each mouse was perfused with ice cold PBS followed by 4% PFA/5% sucrose. The hearts and aortas were removed and analyzed as described below.

### Analysis of Serum Cholesterol and Triglyceride Levels

At the end of the atherosclerosis study blood cholesterol and triglyceride measurements were gathered from serum samples of all mice in each group. A fluorometric assay kit (Cayman Chemical) was used to determine total cholesterol. Samples were diluted 1:200 with assay buffer in duplicates prepared in a 96-well plate. Serum samples were also analyzed for triglyceride levels. A colorimetric assay (Cayman Chemical) allowed for the measurement of fluorescence or absorbance using a SpectraMax Microplate Reader. Standard curves were derived from standard solutions provided in the kit.

### Analysis of Lesion Area of Whole Aortas and Aortic Root Cryosections

Upon dissection of the animal, adventitial fat was cleared away from aorta and branching arteries. The entire aorta was then cut open longitudinally, excised, and pinned to a wax lined dissecting tray. Oil-Red-O Stock (ORO) solution was prepared with 1 g of ORO powder (Sigma-Aldrich) dissolved in 300 mL of 99% isopropanol. A working solution of the stain was prepared fresh by mixing 180 mL of stock solution with 120 mL Milipore water. The solution was allowed to equilibrate for 1 h before filtration with Whatman filter paper into a clean glass bottle. 60% isopropanol was used to wash the pinned aortas which were then completely submerged in ORO working solution for 15 min. The aortas were washed with 60% isopropanol until the stain was no longer visibly being removed (3–4 washes). Aortas were photographed and the images were analyzed to measure plaque area using the ImageJ software.

Fixed hearts were collected from the study mice and mounted in Optimal Cutting Temperature (OCT) compound (Tissue-Tek) which were then frozen at −80°C. Embedded hearts were sectioned in a sagittal orientation through the aortic valve were all three leaflets are visible. Cryo-sectioning was achieved with a cryostat and serial sections of 10 μm increments were made, and five representative sections were selected from each mouse. These sections would be used to visualize lesion area as represented with ORO staining. Newly prepared cross-sections were incubated in PBS for 5 min and then air dried. They were dipped 10 X in 60% isopropanol and then stained with fresh ORO working solution for 15 min. The staining solution was removed and the slides were again dipped 10 X in isopropanol and washed for 5 min under running tap water. Slides were covered using mounting media (Sigma-Aldrich) and dried overnight. Photos were taken of the sections at 5× zoom and the ORO stained area was quantified using ImageJ and represented as a percentage of total aortic area.

### Immunohistochemical Staining of Aortic Root Cryosections

Immunohistochemical (IHC) staining was used to characterize and quantify CHIT1 expression, macrophage populations, necrotic core area, ECM deposition, and collagen formation in WT and CHIT1-OE mouse, aortic cryosections. Slides with mounted cryosections were prepared for IHC by fixing in ice cold, 100% acetone for 1 min and then washed with PBS using dip-style glass chamber. Tissue sections were circled with a PAP pen and then washed with PBS-Tween. Antigen retrieval was carried out using 0.05% trypsin/0.1% calcium chloride solution (trypsin/CA). Slides were incubated with trypsin CA in a humidified chamber at 37°C for 15 min, and allowed to cool at room temperature for 10 min. Antigen retrieval solution was removed and slides were washed three times with PBS-Tween. 0.1% Triton X-100 was then applied for 20 min, removed, and slides were washed again with PBS-Tween.

Hydrogen peroxide block was performed by adding enough 3% hydrogen peroxide to cover each section and incubate until bubbles can no longer be observed emanating from the tissue. Slides were then rinsed with PBS and placed in a dip chamber containing PBS for 2 min. Sections were blocked with 5% donkey serum dissolved in 2% BSA with 1X PBS. Slides were incubated in block solution for 1 h at room temperature.

Primary antibodies (Table 1) for CHIT1 (Santa Cruz Biotechnology) and macrophages (MOMA-2, Abcam) were added to the sections and allowed to incubate overnight at 4°C. Sections were washed with PBS-Tween followed by addition of the horseradish peroxidase (HRP), conjugated secondary antibody. Slides were incubated in a dark humidified chamber for 1 h at room temperature. Sections were washed with PBS-Tween and kept wet until mounted. One drop of reagent a was diluted in 1 mL of distilled water as described in the AEC staining kit instructions (Invitrogen). One drop of reagent be and reagent C were then added and the solution was kept away from light and incubated with the cryosections for 30 min (or until color development is satisfactory). Slides were then rinsed with distilled water. Mayer’s hematoxylin counter stain was applied using a dip chamber for no more than 10 s. The slide was then washed and rinsed under running tap water for 5 min. Next, the slides were put into a dip chamber containing Scott’s Bluing solution for 30 s and dipped once in tap water to wash. Dako fluorescent mounting media was added to the top of each section, which was then covered with a coverslip and stored at 4°C.

Visualization of hyaluronic acid was achieved using biotinylated hyaluronic acid binding protein (BHABP, EMD/Millipore/Calbiochem). After acetone fix, hydrogen peroxide block, and blocking (2% BSA in 1X PBS), streptavidin/biotin blocking was performed according to the instructions provided by the kit (Vector Laboratories): incubate section with Avadin D solution for 15 min at room temperature rinse with PBS-Tween, next incubate section with biotin solution for 15 min at room temperature and rinse again with PBS-Tween. BHABP was diluted 1:100 in blocking solution and applied to sections. These were allowed to incubate at room temperature for 1 h. BHABP was aspirated away and slides were washed 3X with PBS. The streptavidin-conjugated HRP was then applied to the cells for 1 h at room temperature before washing 3X with PBS. AEC chromogen and Mayer’s hematoxylin counter stain was added as described above.

Hyaluronidase negative controls sections were prepared in the same way as sample sections. However, no detection stain was added. Instead, cryosections were incubated with hyaluronidase (StemCell Technologies) for 2 h at room temperature.

Picro-Sirius Red staining was used to visualize collagen in the aortic root. Cryosections were acetone fixed and trypsin/CA antigen retrieval was applied. As described in the instruction manual (Abcam), Picro-Sirius red solution was added to completely cover the section, and incubated for 60 min. The slide was rinsed with two changes of acetic acid solution, and then rinsed once in absolute alcohol. The cryosections were then dehydrated into changes of absolute alcohol and the slide was mounted in resinous mounting media.

### Statistical Analysis

A minimum of three biological replicates run in duplicate or triplicate were analyzed for *in vitro* experiments as indicated. All Data are represented as the average of biological replicates ± SEM. Quantification in each cryosection was carried out by acquiring five images from representative regions of the aortic sinus. Images were analyzed using ImageJ. Statistical analysis was performed using GraphPad Prism 8 software. To determine significance, unpaired, two-tailed Welsh’s *t*-test was performed when comparing experimental and control groups. ^∗^*P* < 0.05, ^∗∗^*P* < 0.005, ^∗∗∗^*P* < 0.0005, ^****^P < 0.0001.

## Results

### Validation of CHIT1 Overexpression

Initial experiments were performed to confirm CHIT1 overexpression in BMDM harvested from CHIT1-OE mice. As mentioned above, CHIT1-Tg mice were crossbred with ldlr^–/–^ mice, thereby providing us with an atherosclerotic, CHIT1 overexpressing mouse model. Moreover, CHIT1 overexpression is achieved using the Cre-Lox system whereby the removal of a LoxP-flanked stop sequence allows for conditional overexpression of CHIT1 in lysozyme producing macrophages (Figure 1A). Conditional overexpression with LysMCre is made possible because the Cre recombinase gene is inserted after the promoter of the LysM gene and as lysozyme is constitutively expressed so too is Cre recombinase, and with that CHIT1.

### CHIT1 Overexpression in Macrophages

To verify the overexpression of CHIT1 in macrophages, BMDM were harvested from both experimental and littermate control animals. Isolated RNA was analyzed with “quantitative” PCR (qPCR). We found that CHIT1 transcription was not affected in control BMDM as the transcript detected was similar to our internal control, GADPH (Figure 1C). CHIT1 transcription was significantly increased in CHIT1-OE BMDM when compared to control BMDM. Cell lysates were prepared from BMDM and visualized using Western blot analysis. Figures 1D,E show protein expression was enhanced in CHIT1-OE BMDM as evidenced by the presence of one distinct band at 50 kDa and one at 39 kDa (per company’s description of CHIT1 antibody, double bands are a result of disulfide bonds) which are characteristic of the predicted molecular weight of both CHIT1 isoforms. The 39 kDa isoform of CHIT1 is a product of the post-translational modification of the 50 kDa isoform and was only visible in CHIT1-OE BMDM. We determined that CHIT1 enzymatic activity is elevated in both cell lysates and the supernatant of CHIT1-OE BMDM as exhibited by the cleavage of 4-MU-conjugated substrates (Figure 1F). Due to the fact that only the 50 kDa isoform of CHIT1 is secreted while the 39 kDa isoform is sequestered in specialized vesicles, we analyzed the supernatant of macrophage cell cultures for the presence of CHIT1. As expected, we found that the 50 kDa, secreted isoform of CHIT1 was robustly expressed in the supernatant of CHIT1-OE BMDM (Figures 1G,H). *N* = 3.

### Overexpression of CHIT1 Modulates Cytokine Expression in Macrophages

**FIGURE 2:**
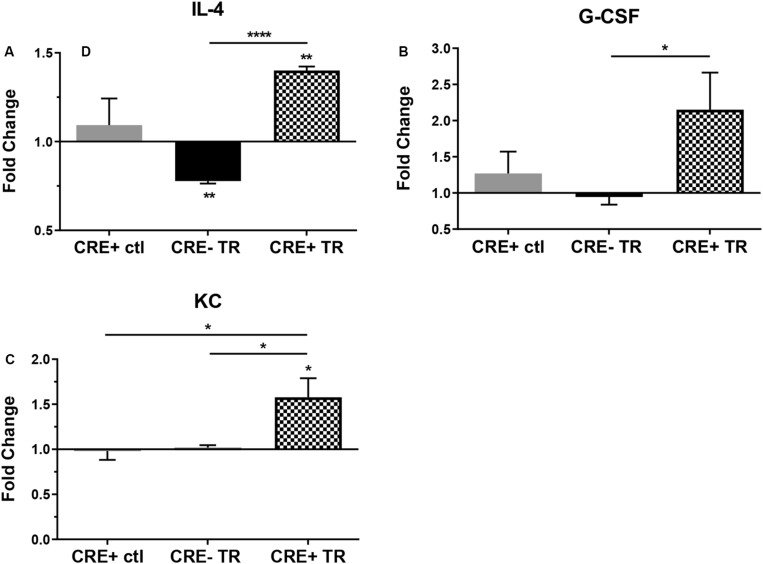
Protein expression in CHIT1-OE BMDM and control BMDM was measured with a mouse cytokine array. Cell lysates were prepared from experimental and control BMDM which were untreated or exposed to 20 ng/mL IFN-γ + 100 ng/mL LPS for 8 h To induce pro-inflammatory activation. Between groups, treated BMDM exhibited a significant increase in protein expression between groups of **(A)** IL-4 (*P*<0.0001), **(B)** G-CSF(*P* = 0.0497), and **(C)** KC (*P* = 0.0426). *N* = 3 biological replicates, each run in duplicate. Values represent fold change in florescence. *P<0.05, ****P<0.0001.

In order to better understand the effects of CHIT1 overexpression on cytokine expression by macrophages, a cytokine antibody array was performed using nitrocellulose membranes spotted with capture antibodies for a variety of cytokines and chemokines. Given that CHIT1 is highly expressed by activated macrophages, the classic combination of IFN-γ and LPS was used to stimulate macrophages by a TLR-2 and TLR-4 respectively. The assay analyzed cell lysates prepared from BMDM, treated with 20 ng/mL IFN-γ + 100 ng/mL LPS (TR). Under these conditions, macrophages are polarized toward an inflammatory phenotype which is reflective of the atherosclerotic plaque environment. Membranes were incubated with cell lysates from both CHIT1-OE BMDM (CRE+) and control BMDM (CRE-) for 8 h prior to application of biotin-labeled detection antibodies. Results in Figures 2A–C showed a significant up regulation of G-CSF, the anti-inflammatory cytokine IL-4, and KC (the murine homolog of IL-8) which is involved in immune cell recruitment, macrophage phagocytosis, parasitic invasion defense, and is expressed during both M1 and M2 inflammatory conditions, *N* = 3.

### Inflammatory Signaling Pathways Are Inhibited by CHIT1 Overexpression

**FIGURE 3:**
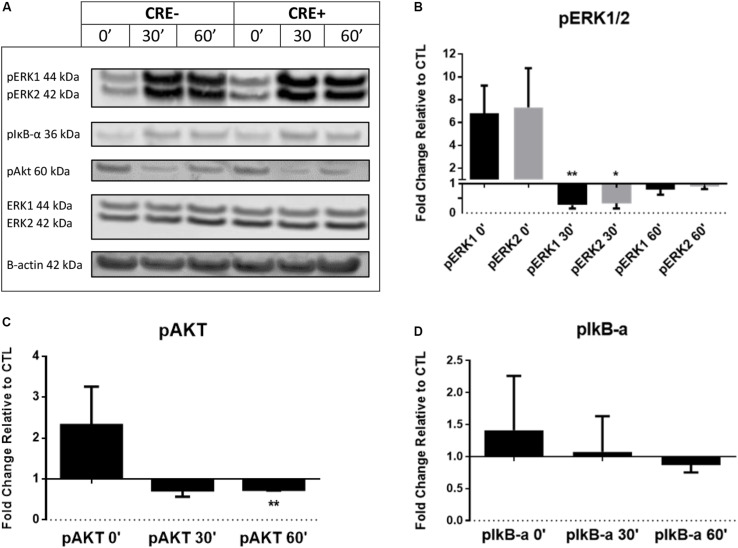
BMDM from CHIT1-OE mice and control animals were treated with 20 ng/mL IFN-γ + 100 ng/mL LPS for 0, 0.5, and 1 h. **(A)** Cell lysates were incubated with florescent antibodies for phosphorylated signaling molecules and expression of phosphorylated proteins was analyzed by Western blotting. Florescence intensity was measured using the Licor Odyssey cLx. All bars represent fold change in densitometric, florescence intensity between CHIT1-OE BMDM and control BMDM. Florescence signal from Western blot of phosphorylated proteins: pIκB-α, pAkt, and pERK1/2 was normalized to control protein and then compared between groups. **(B)** Exposure to pro-inflammatory stimuli significantly depressed pERK1 and pERK2 signaling in CHIT1-OE BMDM after 0.5 h (*P* = 0.0085 and 0.0219 respectively). **(C)** pAkt signaling was significantly different between groups after 1 h when compared to control BMDM (*P* = 0.0020). **(D)** No significant results were obtained in protein expression of pIκB-α. *N* = 3, *P<0.05, **P<0.01.

Phosphorylation is a fundamental component of intracellular signal transduction. To further explore the cell signaling potential of CHIT1, we treated both CHIT1-OE (CRE+) and control BMDM (CRE-) with 20 ng/mL IFN-γ + 100 ng/mL LPS for 0, 0.5, and 1 h. Cell lysates were prepared and analyzed via Western blot for specific phosphorylated signaling molecules: ERK 1/2, Akt, and IκB (Figure 3A). Although not significant between groups or across time points, CHIT1-OE and control BMDM both exhibit increases in pERK 1/2, pAkt, and pIκB at the 0-h time point. This effect, however, was exaggerated in experimental BMDM when compared to controls. pERK1/2 and pAkt was significantly down regulated after 1 and 0.5 h periods, respectively, upon incubation with pro-inflammatory stimuli (Figures 3B,C). Thus, depicting a decrease in phosphorylated signaling in these pathways. This is consistent with diminished transcription of pro-inflammatory cytokines that we observed. Values were quantified from Western blot using densitometric analysis with the Licor Odessey CLx and ImageStudio software. ERK1/2 and β-actin were used as experimental controls, *N* = 3.

### Characterization of Mice Before and After HFD

**FIGURE 4:**
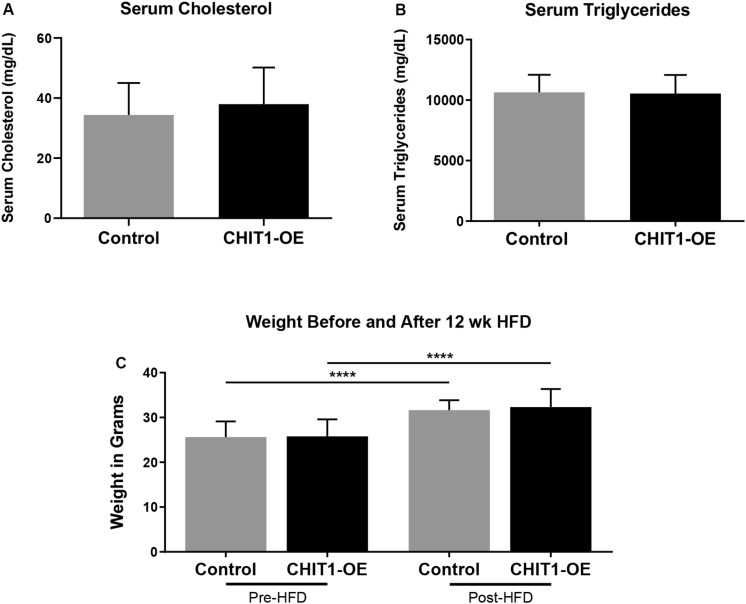
**(A,B)** Measurements of serum cholesterol and triglyceride were taken after 12 weeks of HFD. Colorimetric and fluorometric analysis revealed no significant difference between control mice and CHIT1-OE mice. **(C)** Animals were weighed before and after initiation of HFD, which showed significant weight gain within groups over the 12 week period. However, animal weights were not significantly different when compared between CHIT1-OE and control groups (*P* < 0.0001). *N* = 24 (12 per group), ****P < 0.0001.

Upon termination of the HFD regimen, blood was collected from all study mice in each group prior to being euthanized. Serum cholesterol and triglyceride levels were subsequently measured with fluorometric and colorimetric analysis (Figures 4A,B). No statistically significant difference in cholesterol or triglycerides was detected between experimental and control groups. Measurement of body weight before and after 12 weeks of HFD was also conducted (Figure 4C) and revealed significant weight gain in both post-HFD groups, although no differences observed between groups, *N* = 28 (14 per group).

### CHIT1 Is Highly Expressed in Atherosclerotic Plaques of CHIT1-OE Mice Post-HFD

**FIGURE 5:**
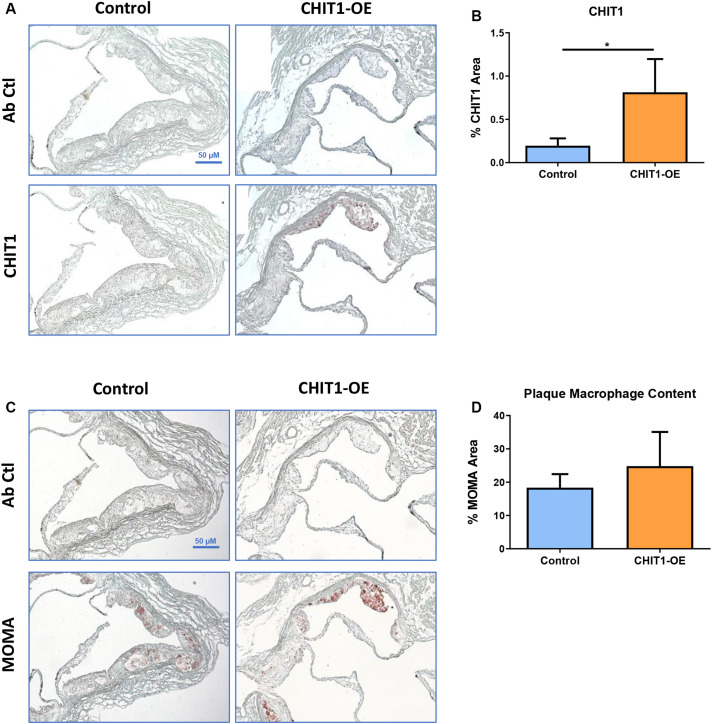
**(A)** Cryosections of the aortic sinus from both CHIT1-OE mice and littermate controls after 12 weeks of HFD were stained with biotinylated antibodies against CHIT1. **(B)** Immunohistological staining for CHIT1 revealed significantly enhanced CHIT1 expression in the atherosclerotic plaques of CHIT1-OE mice when compared to control animals. Quantification represents stained area as a percentage of total plaque area (*P* = 0.0101). **(C)** Serial cryosections of the aortic sinuswere also stained with MOMA to determine macrophage content. **(D)** Analysis of stained area versus % total plaque area. Measurement with ImageJ software showed no significant differences in macrophage content between experimental and control animals (*P* = 0.1982). *N* = 7 per group, *P < 0.05.

Given the importance of macrophages in every stage of atherosclerosis and the fact that CHIT1 is among the most abundantly expressed proteins by activated macrophages, we wished to investigate the presence of CHIT1 in the plaques of our control mice. We also wanted to determine how much more CHIT1 expression was achieved in the lesion of the *CHIT1-OE* mice compared to the control mice. We stained serial cryosections prepared at a thickness of 10 μm of the aortic sinus with a murine CHIT1 antibody (Figure 5A). Ensuing 24 h of incubation, a secondary HRP conjugated chromogen was applied and CHIT1 was visualized using light microscopy. The staining revealed significantly more robust expression of CHIT1 in the atherosclerotic plaques of CHIT1-OE mice in comparison to littermate controls (Figure 5B). CHIT1 staining was quantified using ImageJ software and expressed as the CHIT1 stained area as a percentage of the total plaque area, *N* = 7.

### Macrophage Content in Atherosclerotic Plaques Is Unaffected by CHIT1 Overexpression

Macrophages play multifaceted roles in the development of atherosclerosis. Recruitment and accumulation of macrophages is associated with increased plaque size and weakening of the fibrous cap. We sought to determine whether CHIT1 overexpression in *Ldlr*^–/–^ mice alters macrophage accumulation in atherosclerotic plaques. To this end, slides were prepared from serial cryosections of the aortic sinus at 10 μm increments. Figure 5C depicts samples from each group that were incubated for 24 h with MOMA primary antibody and then and HRP conjugated chromogen was applied. MOMA is a macrophage specific stain and therefore, it was utilized to determine macrophage content and plaques. No significant difference was observed between groups after analyzing percent MOMA stained area relative to total plaque area (Figure 5D), *N* = 7.

### CHIT1 Overexpression in Atherosclerotic Mice Does Not Affect Plaque Area

**FIGURE 6:**
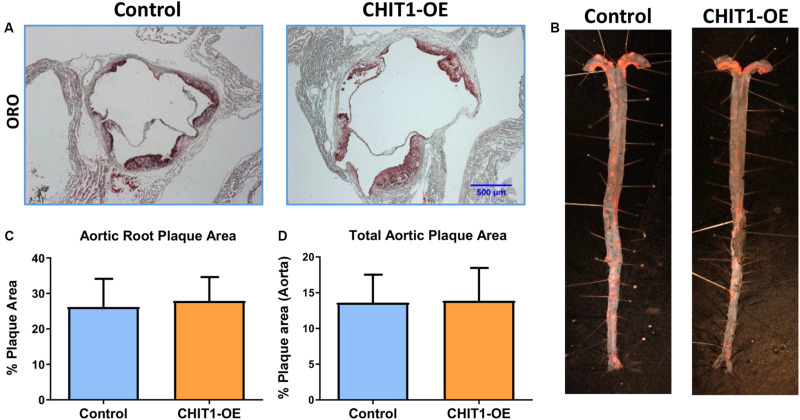
**(A)** Cryosections of the aortic sinus from both CHIT1-OE (CRE+) mice and littermate controls (CRE-) after 12 weeks of HFD were stained with Oil-Red-O to determine plaque size. **(C)** Analysis of plaque area is represented as a function of % total area. Measurement with ImageJ software showed no significant differences in plaque size between experimental and control animals (*P* = 0.5399). **(B)** Whole aortas were removed from both CHIT1-OE and control mice after 12 weeks of HFD. The specimens were pinned and stained with Oil-Red-O. **(D)** No difference was observed between groups upon quantification of plaque area as a percentage of total aortic area (*P* = 0.8731). *N* = 14 per group.

Areas of the cardiovascular system that are subject to high flow volumes and shear stress for vulnerable regions for the development of atherosclerotic plaque. The aortic sinus, aortic arch, and abdominal aorta are widely accepted as being indicative of atherosclerosis *in vivo*. In an effort to determine the effects of CHIT1 overexpression in *Ldlr*^–/–^ mice, mouse hearts were perfused with 4% PFA/5% sucrose before being removed from the animal. Serial cryosections were prepared at a thickness of 10 μm. Samples from both groups were stained with ORO (Figure 6A). Plaque area was not significantly different in CHIT1-OE mice compared to littermate controls and quantification was performed using ImageJ software and measurements are represented as the percentage of stained area in relation to total sinus area. No significant differences in the area of ORO-stained lesion were observed between the control and the overexpressing mice (Figure 6C), *N* = 14.

To further analyze plaque content *in vivo* we also quantified plaque accumulation in the whole aorta. This was accomplished by cleaning and excision of the entire aorta from the aortic arch to the iliac bifurcation. An incision was made longitudinally along the length of the aorta such that it could be separated, pinned, and stained with ORO for 30 min (Figure 6B). Plaque aggregation was measured with ImageJ software and results are represented as percentage of stained area in relation to total aortic area (Figure 6D). As with the aortic sinus lesions, there were no significant differences in the extent of lesion development on the inner surfaces of the aortas between the 2 groups (Figure 5D), *N* = 14.

### CHIT1 Overexpression Alters ECM Content in the Aortic Sinus of *Ldlr*^–/–^ Mice

At sacrifice, mice were perfused with PBS followed by 4% PFA/5% sucrose. The hearts were collected and embedded in OCT compound to be frozen and cryosectioned at a thickness of 10 μm. Serial sections were made at the aortic sinus where three valve leaflets were visible. The heart sections were stained for hyaluronic acid (HA) using a biotinylated hyaluronic acid binding protein (BHABP) and collagen using picrosirius red.

**FIGURE 7:**
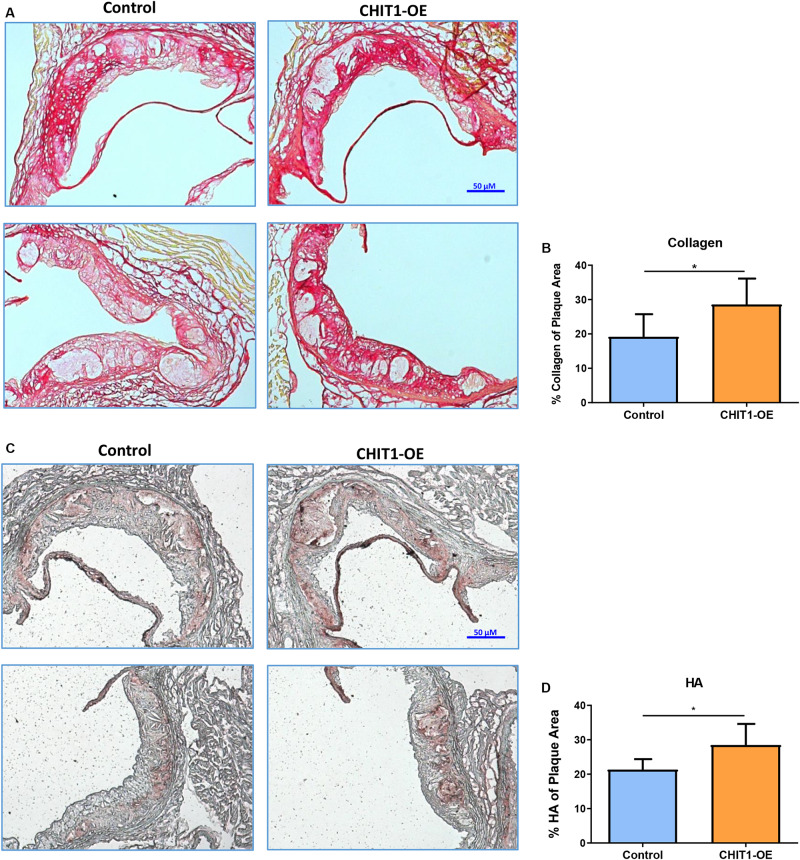
**(A)** Cryosections of the aortic sinus from both CHIT1-OE mice and littermate controls after 12 weeks of HFD were stained with picrosirius red to visualize type I collagen. While collagen appears unorganized and lightly stained, especially on the luminal aspect of necrotic cores in control sections, collagen observed in CHIT1-OE mice presented a more organized “lattice” appearance and was distributed around necrotic cores and specifically on the luminal aspect. **(B)** Quantification of picrosirius red as a percentage of total plaque area exhibited significantly more collagen deposition in CHIT1-OE mice compared to littermate controls (*P* = 0.0289). **(C)** HA stained with BHABP can be seen localized in the periphery of the aortic sinus in control cryosections, whereas HA appears throughout the plaque and within the necrotic cores of CHIT1-OE. HA is extensively visible in plaques of CHIT1-OE mice. Interestingly, HA appears to accumulate within the acellular necrotic core regions. **(D)** Analysis of BHABP staining revealed significantly greater HA content in the aortic sinus of CHIT1-OE mice compared to littermate controls (*P* = 0.0370). *P < 0.05, *N* = 7 per group.

Our data show that the pattern of collagen and HA accumulation had observational differences in distribution between CHIT1*-*OE mice and littermate controls (Figures 7A,C). We found that collagen distribution in plaques from CHIT1-OE mice was most prevalent on the luminal aspect of atherosclerotic plaques and could be found encircling necrotic cores. Picrosirius red staining was significantly more abundant and saturated in CHIT1-OE mice. We also observed necrotic cores being densely surrounded by collagen fibers in aortic sinus sections from CHIT1-OE mice. These characteristics of collagen were not seen in littermate controls. From our *in vivo* analysis, we also determined that HA expression was generally limited to the periphery of atherosclerotic plaques in control mice, while in CHIT1-OE mice HA staining could be seen within almost all aspects of the plaque. Most notably, HA staining colocalized with necrotic regions of the atherosclerotic plaque in CHIT1-OE mice. Stained cryosections were analyzed using ImageJ software to quantify the area of HA and collagen deposition as a percentage of overall plaque area (Figures 7B,D). Both HA and collagen were significantly more abundant in CHIT1-OE mice. *N* = 7.

## Discussion

In previously published work, we reported that CHIT1 mRNA was present in atherosclerotic lesions within the descending aorta of cynomolgus monkeys. CHIT1 mRNA expression was also closely correlated with macrophage infiltration in atherosclerotic plaques of abdominal aortas. Our research further demonstrated that inhibition of CHIT1 with allosamidin promoted atherosclerosis in hyperlipidemic mice (Kitamoto et al., 2013). Here we show *In vitro* evidence using CHIT1-OE macrophages that overexpression of CHIT1 modulates cytokine expression in an anti-inflammatory and migratory manner when subjected to an inflammatory stimulus of IFN-γ with LPS. A non-resolving inflammatory response promotes the development of atherosclerosis through continuous recruitment and activation of immune cells, perhaps most importantly, macrophages. As CHIT1 is abundantly produced by activated macrophages, it is likely that it’s expression contributes to anti-inflammatory cell functions. In developing a *Ldl*^–/–^- CHIT1-OE mouse model we were able to conduct an *in vivo* mouse study to investigate the effects of CHIT1 overexpression in an atherosclerotic animal model. While we were unable to observe significant differences in macrophage content or lesion size, we found significant alterations in plaque morphology. The accumulation and distribution of HA and collagen allude to a possible interaction between CHIT1 and the ECM.

Dysregulation of the innate immune response drives atherogenesis, and the resultant stages of atherosclerosis are largely attributable to the recruitment and infiltration of monocytes into the intimal layer of the vessel wall. Interestingly, we found protein expression of the chemotactic cytokine KC (the murine homolog of IL-8) is significantly upregulated in CHIT1-OE macrophages when treated with inflammatory stimuli. KC and its receptor CXCR2 are secreted by activated macrophages and play a significant role in immune cell trafficking and infiltration of the vessel wall. In an effort to elucidate the mechanism of KC/CXCR2 in atherosclerosis, Boisvert et al. utilized LDLR^–/–^, atherosclerosis-prone mice with KC/GRO-α^–/–^ and CXCR2^–/–^ mice to demonstrate that overexpression of KC/GRO-α and CXCR2 are essential to macrophage accumulation into atherosclerotic lesions. Results also revealed that KC/GRO-α and CXCR2 do not play a critical role in macrophage recruitment into early atherosclerotic lesions (Boisvert et al., 2006). While it is generally accepted that KC participates in development of atherosclerosis, other studies suggest a putative role of IL-8/KC in smooth muscle cell proliferation and recruitment as well as angiogenesis (Yue et al., 1994; Boisvert et al., 1998; Simonini et al., 2000). IL-8 was shown to be a potent macrophage-derived mediator of angiogenesis. Human recombinant IL-8 was administered in the rat cornea where it induced proliferation and chemotaxis of human umbilical vein endothelial cells. Also, blockade of IL-8 by antibodies decreased angiogenic activity of inflamed human rheumatoid synovial tissue macrophages (Koch et al., 1992). Such findings suggest that IL-8 may be involved in tissue repair and wound healing.

IL-4 is a cytokine associated with alternative macrophage activation which potentiates Th2 inflammatory processes. We have demonstrated that IL-4 protein expression is significantly upregulated in CHIT1-OE BMDM. Experimental evidence demonstrates that IL-4 signaling involves SOCS1 to induce M2 macrophage polarization while inhibiting SOCS3 signaling and negatively regulating M1 polarization (Whyte et al., 2011; Qin et al., 2012). IL-4 activation of alveolar macrophages has been shown to promote wound healing in the long after Helmand invasion or during gut inflammation in mice (Bosurgi et al., 2017). And in the context of atherosclerosis, IL-4 attenuated atherosclerosis in several different mouse models (George et al., 2000; King et al., 2002; Davenport and Tipping, 2003). Given that IL-4 and IL-13 signaling includes a pathway requiring both cytokines, and the putative interactions of CHIT1 with IL-13, it is foreseeable that CHIT1 can participate in IL-4/IL-13 signal transduction.

A definitive role for macrophage-derived granulocyte colony stimulating factor (G-CSF) in atherosclerosis has yet to be established. However, several studies suggest protective roles for G-CSF in atherosclerotic animal models. We observed a significant increase in protein expression of G-CSF in BMDM harvested from CHIT1-OE mice compared to WT controls. Experiments with human peripheral blood mononuclear cells (PBMC) illustrated that G-CSF post-transcriptionally inhibits TNF-α secretion from PBMC. Suppression of TNF-α secretion was accomplished without affecting mRNA expression (Kitabayashi et al., 1995). G-CSF also promotes generation of type-1 regulatory T cells (Treg) that secrete the anti-inflammatory cytokine IL-10, and TGF-β1 which is associated with tissue repair, wound healing, and fibrosis (Rutella et al., 2004). Several studies have described a protective role for G-CSF in atherosclerotic mouse and rabbit models. Daily treatment of ApoE deficient mice with G-CSF for 9 weeks resulted in less atheromatous plaque area and a decrease in macrophage infiltration into atherosclerotic lesions. The authors also observed decreased serum cholesterol and LDL, which is suggestive of lipid-related effects of G-CSF (Sinha et al., 2014). In hyperlipidemic and cholesterol fed rabbit models, G-CSF administration prevented progression of atherosclerotic lesions and vascular stenosis, while encouraging plaque stability and reendothelialization. Moreover, it was shown that an appropriate dosage of G-CSF was required for these protective effects to manifest most efficiently (Hasegawa et al., 2006; Matsumoto et al., 2010).

To extend our understanding of the inflammatory mechanisms affected by CHIT1 overexpression, we investigated inflammatory signaling pathways associated with atherosclerosis. Our data revealed significant decrease in pERK1/2 protein expression in BMDM from CHIT1-OE mice compared to littermate controls when exposed to IFN-γ + LPS after 30 min *in vitro*. We also observed that Akt signaling was suppressed in CHIT1-OE BMDM after 60 min of treatment with the same inflammatory stimuli *in vitro*. The ERK1/2 signaling pathway is critical to the expression and secretion of various downstream effectors that mediate inflammation. Experimental evidence demonstrates that LPS stimulation of murine peritoneal macrophages activated ERK1/2 signaling, causing the subsequent induction of TNF-α secretion (Dumitru et al., 2000). IFN-γ stimulation of this pathway in human macrophages induced alterations in the expression of several key genes implicated in the progression of atherosclerosis, such as MCP-1 (Li et al., 2010). Akt signaling has various roles in macrophage function. Deficiency of Akt mediated signal transduction elicits M2 macrophage polarization and negative regulation of the TLR4 signaling pathway (Taganov et al., 2006; Chassin et al., 2010; Li et al., 2015). In relation to atherosclerosis, Zhai et al. (2014) found that inhibition of Akt signaling depressed autophagy *in vitro*, and atherosclerotic mouse models exhibited enhanced plaque stability as well as diminished inflammatory response was promoted by Akt/mTOR-mediated autophagy. As it pertains to ECM content, both ERK1/2 and Akt signaling are involved in the turnover of HA and collagen. The hyaluronic acid receptor (CD44) is a macrophage cell surface receptor responsible for the recognition and phagocytosis of HA. Activation of ERK1/2 and Akt signaling pathways by CD44 results in degradation of HA by hyaluronidase (Culty et al., 1992; Stern and Jedrzejas, 2006; Vachon et al., 2006). Activation of these pathways has also been shown to enhance collagen degradation by matrix metalloproteinases (Cho et al., 2008).

The ECM is an important component for a wide array of cellular functions. In the cardiovascular system, ECM interactions serve as a cell signaling platform and maintain structural integrity of the heart and vasculature. Changes in ECM content related to atherosclerosis has been implicated in fibrous cap formation and plaque stability. Our results demonstrate a significant increase in key ECM components: hyaluronic acid (HA) and collagen in the plaques *CHIT1-OE* mice when compared to littermate controls. HA is a large, hydrophilic glycosaminoglycan (GAG) that is integral to wound healing and tissue remodeling (Litwiniuk et al., 2016). HA catabolism is carried out by enzymes known as hyaluronidases (Hyal) which hydrolyze β(1-4) glycosidic bonds between the alternating moieties of D-glucoronic acid and *N*-acetyl-D-glucosamine. Cleavage of these linkages results in a polydisperse range of HA chain length (Stern and Jedrzejas, 2006). Hyal1 is found in the heart and its expression is dependent on activation of the CD44, cell surface receptor and the subsequent initiation of ERK1/2 and Akt signaling (Gee et al., 2002; Vachon et al., 2006; Harada and Takahashi, 2007; Misra et al., 2015). Degradation of HA by hyaluronidases is upregulated in unstable atherosclerotic plaques, while the formation of a pericellular HA matrix supports smooth muscle cell proliferation and migration possibly enhancing plaque stability (Evanko et al., 1999; Bot et al., 2010). It should be noted that no endogenous substrate for CHIT1 has been identified in vertebrates. However, studies have demonstrated that the DG42 gene in Xenopus and mouse models produces chitin-oligosaccharides as a precursor to HA chains (Meyer and Kreil, 1996; Semino et al., 1996). Also, due to the structural similarity of HA to its native substrate; chitin, the CHIT1 binding domain is capable of interactions with HA (Ujita et al., 2003; Crasson et al., 2017). It may be possible that chitin interactions with HA prevent its degradation, thereby promoting plaque stability.

Like HA, collagen contributes to the mechanical strength of atheromatous plaques. Collagen comprises up to 60% of the total plaque protein and engages a multitude of cell functions. Modulation of macrophage behavior, smooth muscle cell proliferation/migration, and plaque reinforcement exemplify the variety of functions and cell types that collagen can influence (Koyama et al., 1996; Rocnik et al., 1998; Wesley et al., 1998). CHIT1 has also been implicated in collagen production as evidenced by fibrotic diseases in the liver and lungs. Interstitial lung disease with pulmonary fibrosis is representative of pulmonary systemic sclerosis. CHIT1^–/–^ murine models demonstrate reduced fibrosis, and *in vitro* studies showed that CHIT1 interactions with TGF-β1 augment the expression of both TGF-β 1 and 2 receptors and subsequent TGF-β-induced ERK activation (Lee et al., 2012). Human Kupffer cells were also implemented to investigate the role of CHIT1 in nonalcoholic steatohepatitis. This data highlighted the ability of CHIT1 to activate hepatic stellate cells which, in turn, results in the overproduction of collagen and ultimately hepatic fibrosis (Malaguarnera et al., 2006). Collagen degradation occurs by the action of matrix metalloproteinases (MMP) and several studies have demonstrated the presence and proteolytic activity of MMPs in vulnerable atherosclerotic plaques (Newby, 2008; Newby et al., 2009; Jabłońska-Trypuć et al., 2016).

In our assessment of plaque morphology, despite having no difference in macrophage content or size, collagen and hyaluronic acid deposition was markedly different between experimental and control animals. *CHIT1-OE* mice displayed hyaluronic acid staining throughout the plaque and around necrotic cores, whereas aortic sections from control mice showed accumulation of hyaluronic acid around the periphery of the vessel. Collagen staining revealed substantial accumulation on the luminal aspect of necrotic cores in CHIT1-OE animals. In contrast, collagen distribution in control animals did not show any particular patterning in atherosclerotic plaques. Taken together, overexpression of CHIT1 by macrophages augments ECM biosynthesis and organization in such a way that enhances plaque stability in ldlr^–/–^ mouse models.

The data presented here suggest a novel, nonenzymatic role for CHIT1 in inflammation and atherosclerosis. Results generated from this work demonstrate that, *in vitro*, CHIT1 modulates macrophage transcription and protein expression of chemokines and cytokines that are central to inflammation and atherosclerosis. We also provide evidence of alterations in macrophage cells signaling pathways. And lastly, analysis of aortic sinus cryosections exhibit significant differences in prevalence of HA and collagen as well as localized distribution between control mice and *CHIT1-OE* mice. As mentioned before, various studies have demonstrated nonenzymatic interactions involving CHIT1, inflammation, and tissue repair. Given our results, it is possible that CHIT1 influences macrophage behavior in such a way that is atheroprotective in mitigating inflammation and enhancing plaque stability.

## Limitations

Although we have described a putative role for CHIT1 as it pertains to plaque stability, our experimentation did not include several key aspects that could further validate our findings. For example, future studies should focus on interaction between CHIT1 and HA (both structurally and metabolically), quantifying fibrous cap thickness and smooth muscle cell content, a measure of plaque vulnerability/stability index, and histological examination of other organs that are prone to fibrosis.

## Data Availability Statement

All datasets generated for this study are included in the article/supplementary material.

## Ethics Statement

The animal study was reviewed and approved by the University of Hawaii IACUC.

## Author Contributions

JY, SM, SK, B-HL, and WB contributed to the conception and design of the study. MA and SK contributed to biochemical analysis of samples. JY, SM, WR, JG, and JI contributed to the design and application of experimental procedures. SM and SK contributed to statistical analysis. JY wrote the first draft of the manuscript. SM, SK, and WB wrote sections of the manuscript. All authors contributed to manuscript revisions, read, and approved the submitted version.

## Conflict of Interest

The authors declare that the research was conducted in the absence of any commercial or financial relationships that could be construed as a potential conflict of interest.
